# Core-shell Au@Pd nanoparticles with enhanced catalytic activity for oxygen reduction reaction *via* core-shell Au@Ag/Pd constructions

**DOI:** 10.1038/srep11949

**Published:** 2015-07-06

**Authors:** Dong Chen, Chengyin Li, Hui Liu, Feng Ye, Jun Yang

**Affiliations:** 1 State Key Laboratory of Multiphase Complex Systems, Institute of Process Engineering, Chinese Academy of Sciences, Beijing 100190, China; 2 University of Chinese Academy of Sciences, No. 19A Yuquan Road, Beijing 100049, China

## Abstract

Core-shell nanoparticles often exhibit improved catalytic properties due to the lattice strain created in these core-shell particles. Herein, we demonstrate the synthesis of core-shell Au@Pd nanoparticles from their core-shell Au@Ag/Pd parents. This strategy begins with the preparation of core-shell Au@Ag nanoparticles in an organic solvent. Then, the pure Ag shells are converted into the shells made of Ag/Pd alloy by galvanic replacement reaction between the Ag shells and Pd^2+^ precursors. Subsequently, the Ag component is removed from the alloy shell using saturated NaCl solution to form core-shell Au@Pd nanoparticles with an Au core and a Pd shell. In comparison with the core-shell Au@Pd nanoparticles upon directly depositing Pd shell on the Au seeds and commercial Pd/C catalysts, the core-shell Au@Pd nanoparticles *via* their core-shell Au@Ag/Pd templates display superior activity and durability in catalyzing oxygen reduction reaction, mainly due to the larger lattice tensile effect in Pd shell induced by the Au core and Ag removal.

Oxygen reduction reaction (ORR) is the key catalytic reaction occurred at the cathode electrode of direct liquid (methanol, ethanol, or formic acid) fuel cells, which have garnered sustained research interest due to their desirable features as energy suppliers for mobile and portable power devices, e.g. relatively small environmental footprint, compact system design, high energy conversion efficiency and higher volumetric energy densities^1^,^2^,^3^,^4^,^5^. Currently platinum (Pt)-based nanomaterials are the most effective electrocatalysts to facilitate ORR in direct liquid fuel cells^6^,^7^,^8^. However, the high cost of precious Pt metal and its instability as ORR electrocatalysts at cathode enormously limit the commercialization of direct liquid fuel cells^9^,^10^. Fortunately, current studies have found that the relatively inexpensive Pd-based nanomaterials have great potential to substitute for Pt as the cathodic catalysts of direct liquid fuel cells^6^,^8^,^11^. Specifically, for direct methanol fuel cell (DMFC), the Pd-based ORR electrocatalysts could be hardly affected by methanol crossover compared with Pt catalysts^12^. Therefore, extensive research efforts have been devoted towards the development of Pd-based nanomaterials for oxygen reduction at the cathode of direct liquid fuel cells^8^,^12^,^13^,^14^. Unfortunately, upon the volcano plot obtained by Nørskov and coworkers^15^, Pd has much lower intrinsic ORR catalytic activity compared with that of Pt due to its inappropriate d-band center, which may weaken the adsorption of oxygen on the catalyst surface, resulting in poor performance in catalyzing oxygen reduction^16^,^17^. Therefore, for the practical use of Pd-based nanomaterials at the cathode of direct liquid fuel cells, it is necessary to maximize the activity of them by engineering their structure, morphology and/or composition.

Bimetallic nanoparticles with a core-shell structure are a type of conventional heterogeneous nanomaterials. The complex electron interaction between the two electron-rich elements and the lattice strain created in these core–shell particles could modify the surface electronic properties of the nanoparticles^18^,^19^. Therefore, core-shell nanoparticles often exhibit improved catalytic properties compared to their alloyed counterparts or to mixtures of monometallic nanoparticles^20^,^21^. For example, as demonstrated by Tsung and co-workers, the single-crystalline core-shell Au@Pd octahedra exhibit the enhanced catalytic activity for oxidation of carbon monooxide (CO) and formic acid due to the lattice strain, which is induced by lattice mismatch between the Au core and Pd shell, owing to the differences in their lattice parameters^22^.

In this work, we report the synthesis of bimetallic Au-Pd nanoparticles with a core-shell construction and investigate their electrocatalytic properties toward ORR. The novelty of this work lies with the synthetic approach. Instead of direct deposition of Pd shell on the Au seeds, we use core-shell Au@Ag/Pd nanoparticles with an Au core and an alloy Ag/Pd shell as an intermediate template to form the final Au@Pd products with a core-shell structure. In this strategy, core-shell Au@Ag nanoparticles with an Au core and an Ag shell are firstly prepared by reducing the Ag^+^ precursors in the presence of pre-synthesized Au seed particles in oleylamine. Then the pure Ag shells are converted into the shells made of Ag/Pd alloy by galvanic replacement reaction between the Ag shells and Pd^2+^ precursors. Subsequently, the core-shell Au@Ag/Pd nanoparticles are agitated with saturated aqueous NaCl solution for the removal of Ag component from the Ag/Pd alloy shells, leading to the formation of final Au@Pd products with remained core-shell structure. We would demonstrate that the core-shell Au@Pd nanoparticles as-prepared have superior activity and durability for ORR in comparison with the core-shell Au@Pd nanoparticles produced by directly depositing Pd shell on the Au seeds and commercial Pd/C catalysts from Johnson Matthey (JM). In addition to the Au core, the Ag removal from the alloy shell may induce additional lattice strain on the remaining Pd shell. The sufficient lattice strain imposed by the Au core and Ag removal, which could tailor the d-band center of Pd shell, may account for the enhanced ORR performance of core-shell Au@Pd nanoparticles.

## Result and Discussion

**Figure 1:**
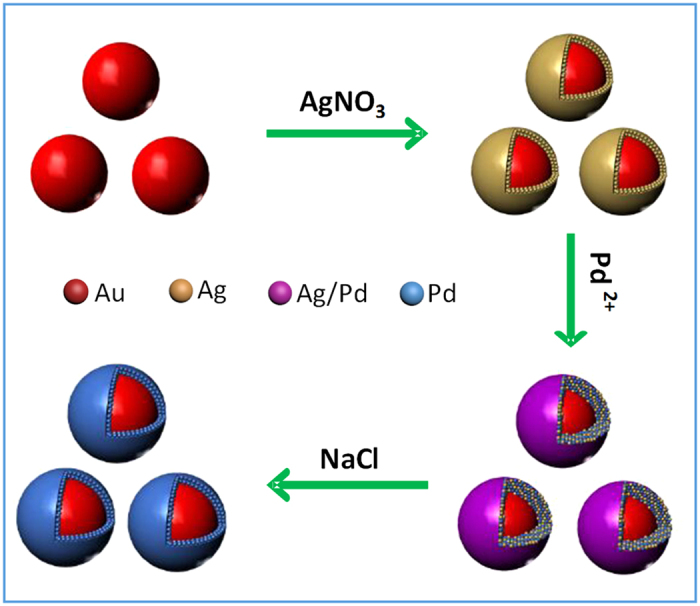
Synthetic strategy. Schematic illustration to show the synthesis of core-shell Au@Pd-I nanoparticles using core-shell Au@Ag/Pd with an Au core and an Ag/Pd alloy shell as intermediate template.

Figure 1 is the schematic illustration to show the synthesis of core-shell Au@Pd-I nanoparticles through the removal of Ag from core-shell Au@Ag/Pd nanoparticles with an Au core and an Ag/Pd alloy shell. Firstly, The Au seeds were synthesized by oleylamine reduction of HAuCl_4_·3H_2_O, where the oleylamine also serves as the stabilizer of Au seed particles. The TEM image of the Au seeds as-prepared in oleylamine is shown in Fig. S1a of Supplementary Information (SI). As displayed, these Au seed particles are spherical, nearly dispersed, and have an average diameter of 11.3 nm. The HRTEM image (SI Fig. S1b) illustrates the lattice planes in these nanoparticles, showing an interplanar spacing of ca. 0.24 nm, which corresponds to the (111) planes of face-centered cubic (fcc) Au (JCPDS Card File 040784)^23^. Then upon the reduction of AgNO_3_ in the presence of Au seeds in oleylamine, core-shell Au@Ag nanoparticles with an Au core and an Ag shell are formed. As shown by SI Fig. S2a, b and c for the TEM, HRTEM and STEM images, respectively, these core-shell Au@Ag particles are spherical in shape and have an average diameter of ca. 12.5 nm, suggesting a thin Ag shell (ca. 0.6 nm) has been deposited on the surface of Au seed particles. The STEM-EDX analysis (SI Fig. S2e) of an arbitrary single particle in SI Fig. 2c illustrates that the particles are indeed composed of Au and Ag. The formation of core-shell nanoparticles is first suggested by brightness contrast between the core and shell regions (SI Fig. S2a), and then confirmed by the element profiles (SI Fig. S2d) of an arbitrarily chosen single particle obtained in the STEM mode (SI Fig. S2c), which reveal that the Ag signal is present throughout the particle whereas the Au signal is concentrated only in the core region. In addition, as shown by SI Fig. S3, the as-prepared core-shell Au@Ag nanoparticles show two ultraviolet absorption bands centered at 428.0 and 500.8 nm, respectively, which are attributed to the surface plasmon resonance of Ag and Au component in core-shell particles. In reference to that of Au seed particles, the blue-shift (ca. 24.2 nm) of the Au surface plasmon band in the core-shell Au@Ag nanoparticles demonstrates the influence of Ag shell on the optical property of Au core^24^. Because of the very similar lattice parameters for Au and Ag^25^, the XRD pattern (SI Fig. S4) of core-shell Au@Ag nanoparticles shows a homogeneous phase.

**Figure 2:**
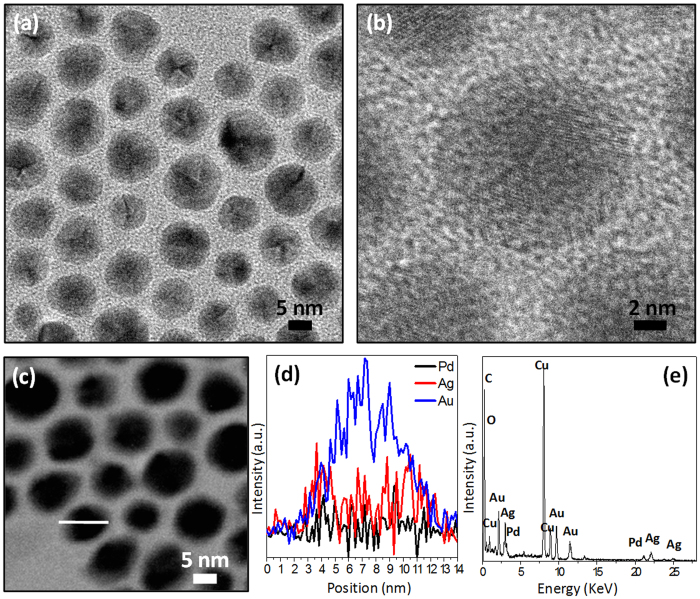
Core-shell Au@Ag/Pd nanoparticles. TEM image (**a**), HRTEM image (**b**), STEM image (**c**), elemental profiles in STEM mode (**c,d**) and STEM-EDX analysis (**c,e**) of core-shell Au@Ag/Pd prepared via galvanic replacement reaction between Ag shell of core-shell Au@Ag nanoparticles and Pd(acac)_2_ precursors.

In the strategy developed in this work, the preparation of core-shell Au@Ag/Pd nanoparticles with an Au core and an Ag/Pd alloy shell is an important step preceding the fabrication of Au@Pd-I nanoparticles. The core-shell Au@Ag/Pd nanoparticles were synthesized *via* galvanic replacement reaction between the Ag shell of core-shell Au@Ag nanoparticles and Pd^2+^ precursors. The replacement reaction between Ag shell and Pd(acac)_2_ can be described as 2Ag + Pd^2+^ → Pd + 2Ag^+^, leading to the conversion from pure Ag shell into an alloy shell consisting of Ag and Pd^26^. The representative TEM, HRTEM, and STEM images of the core-shell Au@Ag/Pd nanoparticles are shown in Fig. 2a–c, respectively. As displayed, the particles synthesized via galvanic replacement reaction remain the spherical shape and average size of their core-shell Au@Ag templates. The STEM-EDX analysis (Fig. 2e) of an arbitrary single particle in Fig. 2c demonstrates that the particles prepared from core-shell Au@Ag nanoparticles *via* replacement reaction are composed of Au, Ag and Pd components. The formation of core-shell nanoparticles with an Au core and an Ag/Pd alloy shell is again confirmed by the elemental profiles of a single particle in the STEM mode. As shown in Fig. 2d, the signal of Au is confined to core region whereas the Ag and Pd signals are uniformly distributed throughout the nanoparticles. Further, in comparison with that of the core-shell Au@Ag nanoparticles, the absorption band of Ag for core-shell Au@Ag/Pd has a blue-shift of ca. 25 nm (SI Fig. S3), which is also an indirect evidence to indicate the conversion of the chemical composition in the shell region^27^. In addition, compared with those of core-shell Au@Ag nanoparticles, a slight shift to higher angles is observed in the XRD pattern of the core-shell Au@Ag/Pd nanoparticles (SI Fig. S4), which could be attributed to the smaller lattice parameter of Pd than that of Ag (0.390 nm for Pd and 0.409 nm for Ag)^28^.

**Figure 3:**
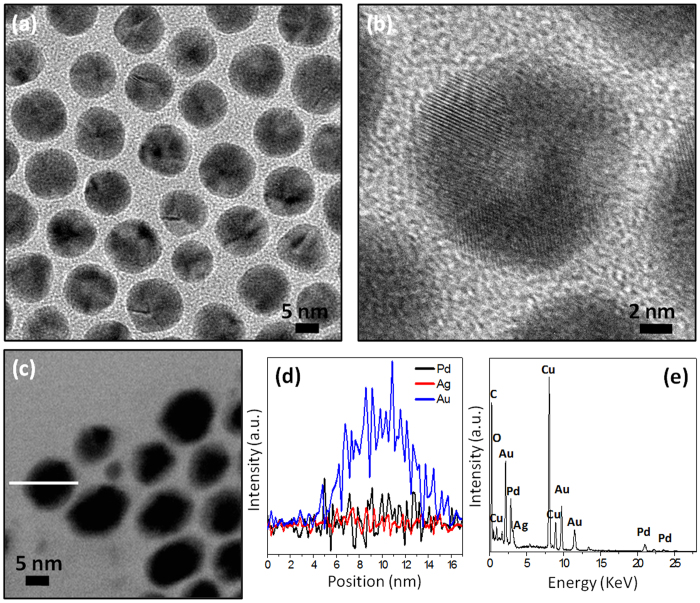
Core-shell Au@Pd-I nanoparticles. TEM image (**a**), HRTEM image (**b**), STEM image (**c**), elemental profiles in STEM mode (**c,d**) and STEM-EDX analysis (**c,e**) of core-shell Au@Pd-I prepared using core-shell Au@Ag/Pd nanoparticles as parent template.

It has been well known that the Ag nanoparticles could be etched by Cl^−^ anions and dissolved O_2_ in solution^29^,^30^. Therefore, after aging the mixture of core-shell Au@Ag/Pd colloidal solution in toluene and saturated aqueous NaCl solution for 24 h under vigorous stirring, the Ag component in the alloy Ag/Pd shell of core-shell Au@Ag/Pd nanoparticles is etched into Ag^+^ cations, which subsequently react with Cl^−^ anions to form AgCl dissolved in the saturated NaCl solution. The elimination of Ag from Ag/Pd alloy shell results in the formation of core-shell Au@Pd-I nanoparticles. As displayed by Fig. 3a,b for the TEM and HRTEM images, the treatment with saturated NaCl solution would not lead to apparent changes in size and morphology of core-shell Au@Ag/Pd nanoparticles. The STEM-EDX analysis (Fig. 3e) of the arbitrarily chosen single particle in Fig. 3c illustrates the significant abatement of Ag component in core-shell Au@Ag/Pd nanoparticles after treatment with saturated NaCl solution, indicating the successful removal of Ag from Ag/Pd alloy shell region. However, as evinced by Fig. 3d for the elemental profiles, which shows that Au still stays at the core region, while Pd is distributed in the periphery of the particles, the core-shell structure is maintained after treatment with saturated NaCl solution. The trace of Ag detected in core-shell Au@Ag/Pd nanoparticles after treatment with saturated NaCl solution by the EDX analyzer in STEM mode (Fig. 3d,e) might be attributed to the adsorption of AgCl on the surface of the remaining core-shell Au@Pd-I particles. Moreover, as observed in SI Fig S3, the disappearance of adsorption band of Ag in core-shell Au@Ag/Pd nanoparticles after treatment with NaCl solution also illustrates the removal of Ag component from the Ag/Pd alloy shell. It should be noted that because the amount of Au in core-shell Au@Ag/Pd and Au@Pd-I nanoparticles is dominant, their XRD pattern is quite analogous, as displayed by SI Fig. S4.

These core-shell Au@Pd nanoparticles are attractive as electrocatalysts for ORR application due to a welcome feature in the core-shell systems: the lattice strain effect of the Au core on the deposited Pd shell. When a thin Pd layer is deposited on the surface of Au seed particles with relatively larger lattice parameters, the interplanar spacing of Pd would be stretched to match the lattice plane of Au for its epitaxial growth on the Au cores. The tensile strain in the Pd shell imposed by the Au core could lead to the upward shift of the Pd d-band center and therefore change the electronic properties of the surface, which are closely associated with the reactivity of the core-shell particles^16^,^31^. To investigate the influence of the Ag removal from the Ag/Pd alloy shell on the lattice tensile effect in the remaining Pd shell, we also prepared core-shell Au@Pd-II nanoparticles by direct growth of thin Pd layer on the Au seed particles in oleylamine for comparison. The TEM image, HRTEM image, and EDX analysis as well as the UV-visible spectrum of the as-prepared Au@Pd-II are displayed in SI Fig. S5a,b,c, and SI Fig. S3, respectively. As indicated, both the average size (ca. 13 nm) and the spherical morphology are comparable with those of Au@Pd-I nanoparticles, and this validates the comparison between the two different core-shell nanoparticles.

**Figure 4:**
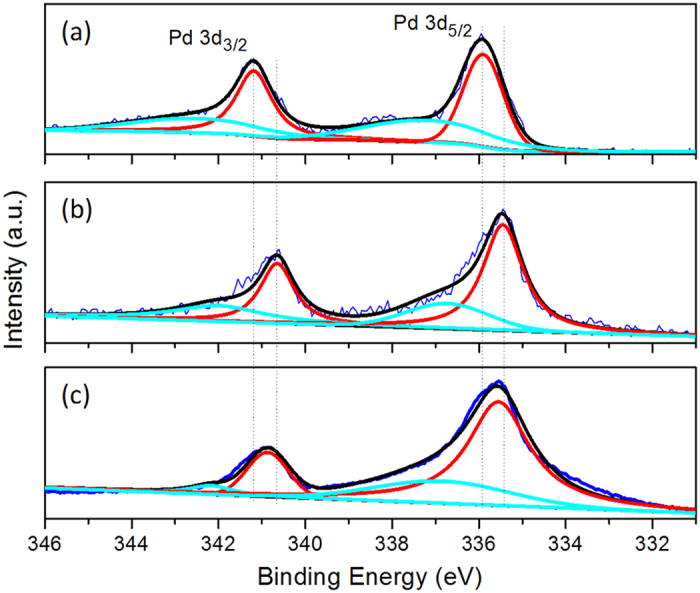
XPS spectra. The 3d XPS spectra of Pd in commercial Pd/C catalysts (a), core-shell Au@Pd-I (**b**), and core-shell Au@Pd-II (**c**), respectively.

As illustrated by SI Fig. S4, the difference in the XRD patterns of core-shell Au@Pd-I and Au@Pd-II is not apparent due to the dominant amount of Au in these core-shell particles. Fortunately, the different tensile effect in the Pd layer of core-ahell Au@Pd-I and Au@Pd-II could be experimentally identified by the Pd 3d XPS spectra. When the tensile strain occurs, the width of the d-band and the energy of its center would be altered. Hence the overlap changes although the degree of d-band filling remains the same. The atoms are pulled apart and the average coordination number decreases, leading to the decreased overlap of the d orbitals, and consequently the band narrows. The center of the d-band moves up in order to maintain the same filling degree. Due to closer to the Fermi level, the d-electrons have become more instable^32^. As the density of states (DOS) at the Fermi level increases, it is easier to “ionize” the metal, resulting in the decrease of Pd XPS binding energies, which are related to the ionization of these metal atoms^33^. As shown in Fig. 4, the XPS 3d spectra of commercial Pd/C, core-shell Au@Pd-I, and core-shell Au@Pd-II were analyzed. The Pd 3d spectra can be deconvoluted into two pairs of doublets. The more intense doublet (at 335.9 and 341.2 eV for Pd/C, 335.4 and 340.7 eV for Au@Pd-I, 335.5 and 340.8 eV for Au@Pd-II) corresponded to Pd at zero valent state, while the second and weaker doublet, with binding energies higher than those of zero valent Pd metal could be assigned to Pd at oxidized state^34^,^35^. Compared with the Pd 3d_5/2_ and 3d_3/2_ binding energies of commercial Pd/C catalysts, an appreciable shift to lower values is observed in the core-shell Au@Pd-I and Au@Pd-II nanoparticles. Moreover, the shift is larger in core-shell Au@Pd-I than that in core-shell Au@Pd-II, indicating that besides Au core, the Ag removal from the Ag/Pd alloy shell could induce additional lattice tensile strain on the remaining Pd shell, which therefore would affect the catalytic reactivity of Pd shell in further degree.

The core-shell Au@Pd-I and Au@Pd-II nanoparticles were loaded on Vulcan carbon support and tested for their electrocatalytic properties for the ORR at room temperature. The loading of the relevant particles on carbon was fixed at 20 wt% of Pd in order to be comparable to the commercial catalyst (Pd/C from JM, labeled as Pd/C-JM, 20 wt% Pd NPs with average size of 3.2 nm on Vulcan XC-72 carbon support), which was used to benchmark the catalyst performance.

**Figure 5:**
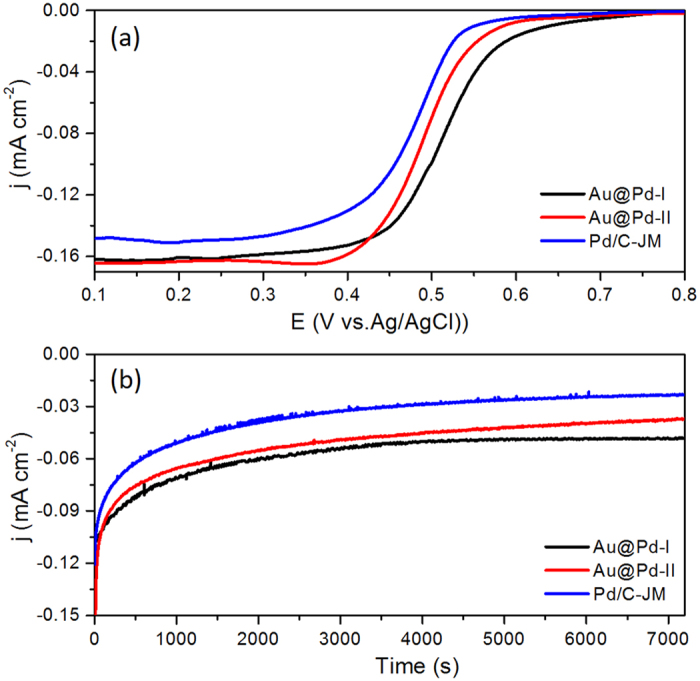
Electrochemical measurements. Negative-going linear sweep voltammograms (**a**) and chronoamperograms at 0.45 V (**b**) of core-shell Au@Pd-I nanoparticles, core-shell Au@Pd-II nanoparticles, and commercial Pd/C-JM catalysts in O_2_ saturated 0.1 M HClO_4_ electrolyte at a scan rate of 10 mV s^−1^ and a rotating rate of 1600 rpm.

The CO stripping voltammograms of core-shell Au@Pd-I, Au@Pd-II, and Pd/C-JM shown in SI Fig. S6 were used to determine the electrochemically active surface areas (ECSAs) of the catalysts^36^. The ECSAs normalized by the mass of Pd are 52.5 m^2^ g^−1^, 58.3 m^2^ g^−1^ and 64.7 m^2^ g^−1^ for core-shell Au@Pd-I, Au@Pd-II NPs, and Pd/C-JM, respectively. The catalytic activities of core-shell Au@Pd-I, Au@Pd-II, and Pd/C-JM catalysts for ORR were then examined and compared. Fig. 5a shows the negative-going linear sweep voltammograms (LSVs) in the potential range from 0.8 to 0.1 V for core-shell Au@Pd-I, core-shell Au@Pd-II and Pd/C-JM catalysts in oxygen-saturated 0.1 M HClO_4_ electrolyte at room temperature. The current densities in the voltammograms are normalized by the ECSAs of the catalysts. As observed, the half-wave potentials for core-shell Au@Pd-I, Au@Pd-II, and Pd/C-JM catalysts are 514, 491, and 478 mV, respectively, and both the core-shell Au@Pd-I and Au@Pd-II nanoparticles exhibit half-potential more positive than that of Pd/C-JM catalysts, indicating that the core-shell Au@Pd nanoparticles have higher catalytic activity for ORR in comparison with that of commercial Pd/C catalysts. In addition, the core-shell Au@Pd-I nanoparticles have the highest catalytic activity toward ORR, which is 23 mV greater than that of core-shell Au@Pd-II nanoparticles. The long-term performance of core-shell Au@Pd-I, core-shell Au@Pd-II nanoparticles, and commercial Pd/C-JM catalyst for ORR was illustrated by the chronoamperograms in Fig. 5b. The slower rate of decay for the Au@Pd-I nanoparticles also indicates their superior stability to the other two Pd-based catalysts.

The observed enhanced catalytic activity for ORR of core-shell Au@Pd nanoparticles could be resulted from the reasonably lattice strain effect in the core-shell structured nanoparticles. An active Pd-based ORR electrocatalyst should have an appropriate d-band center to balance both the bond-breaking and bond-making steps in the common ORR process^37^,^38^,^39^. In core-shell Au@Pd nanoparticles, the upward shift of Pd d-band center due to the tensile strain would strengthen the adsorption of oxygen on the surface of Pd catalysts and promote the O-O band rupture in ORR^40^,^41^. Specifically, the additional tensile effect induced by Ag removal in core-shell Au@Pd-I nanoparticles may more sufficiently balance the bond-breaking and bond-making steps of the ORR process, thus offering the highest ORR catalytic activity among the three catalysts examined.

In summary, we reported the design and synthesis of the bimetallic Au-Pd nanosystems with a core-shell construction to enhance their electrocatalytic activity for room-temperature oxygen reduction reaction. This strategy involves the preparation of core-shell Au@Ag/Pd nanoparticles with an Au core and an Ag/Pd alloy shell, and the removal of Ag component from the alloy shell region using saturated NaCl solution. The lattice tensile strain in the Pd shell imposed by the Au core and Ag removal balance the bond-breaking and bond-making steps in the ORR process, rendering the core-shell Au@Pd nanoparticles as-prepared to display superior ORR activity and durability at room temperature. The concept developed in this work may be extended to explore the fabrication of other bimetallic core-shell structures with favorable lattice strain effect for given technical applications.

## Methods

### General materials

Silver nitrate (AgNO_3_, ACS reagent, ≥99.0%), palladium(II) acetylacetonate (Pd(acac)_2_, 99%), gold(III) chloride trihydrate (HAuCl_4_·3H_2_O, ACS reagent, ≥49.0% Au basis), oleylamine (70%, technical grade) and Nafion 117 solution (5% in a mixture of lower aliphatic alcohols and water) were purchased from Sigma-Aldrich. Ethanol (>99.7%), methanol (>99%), toluene (>99.5%) and perchloric acid solution (70%) were purchased from Beijing Chemical Works. Vulcan XC-72 carbon powders (XC-72C with BET surface area of 250 m^2^ g^−1^ and average particle size of 40 ∼ 50 nm) were purchased from Cabot. Commercial Pd/C catalyst was purchased from Johnson Matthey (JM). All chemicals were used as received. Deionized water was distilled by a Milli-Q Ultrapure-water purification system. All glassware and Teflon-coated magnetic stirring bars were cleaned with *aqua regia*, followed by copious rinsing with deionized water before drying in an oven.

### Synthesis of core-shell Au@Ag and Au@Ag-Pd nanoparticles

For the synthesis of core-shell Au@Ag nanoparticles, 0.1 mmol of HAuCl_4_·3H_2_O was added to 10 mL of oleylamine in a three-necked flask fitted with a condenser and a stir bar. The solution was heated to 150 °C and kept at this temperature for 1 h for the reduction of Au^3+^ by oleylamine, which also serves as the capping agent. Subsequently, 0.04 mmol of AgNO_3_ was swiftly added to the Au seed solution under vigorous stirring at 150 °C, and the mixture was continuously heated at 150 °C for 2 h for the formation of Au@Ag nanoparticles with a core-shell structure. The as-prepared core-shell Au@Ag nanoparticles were used as sacrificial templates to form core-shell Au@Ag/Pd nanoparticles with an Au core and an Ag/Pd alloy shell *via* the galvanic replacement reaction between the Ag shell and Pd^2+^ precursors. In detail, 0.015 mmol of Pd(acac)_2_ was swiftly added to the core-shell Au@Ag colloidal solution in oleylamine at 150 °C under vigorous stirring, and the mixture was kept at 150 °C for another 2 h to achieve the conversion from pure Ag shell in core-shell Au@Ag nanoparticles into Ag/Pd alloy shell in core-shell Au@Ag/Pd nanoparticles. After reaction, the core-shell Au@Ag/Pd nanoparticles thus obtained were recovered by precipitation with methanol, followed by centrifugation and washing with methanol, and then re-dispersed in 10 mL of toluene.

### Synthesis of core-shell Au@Pd nanoparticles using core-shell Au@Ag/Pd nanoparticles as parent templates

For the synthesis of core-shell Au@Pd nanoparticles, labeled as Au@Pd-I, using core-shell Au@Ag/Pd nanoparticles as parent templates, the core-shell Au@Ag/Pd nanoparticles dispersed into toluene were mixed with 20 mL of saturated aqueous NaCl solution, and the mixture was agitated for 24 h at room temperature for the complete removal of Ag component from the Ag/Pd alloy shell region of the core-shell particles. Afterwards, the mixture was left to stand for the complete separation of the two immiscible phases, and then the upper toluene phase containing core-shell Au@Pd-I nanoparticles was collected.

### Synthesis of Au@Pd nanoparticles by the direct growth of Pd shell on the surface of Au seeds

Typically, 0.1 mmol of HAuCl_4_·3H_2_O was dissolved in 10 mL of oleylamine placed in a three-necked flask equipped with a condenser and a stir bar. The solution was heated to 150 °C and kept at this temperature for 1 h for the complete reduction of Au^3+^ ions by oleylamine. Then 0.015 mmol of Pd(acac)_2_ was swiftly added to the colloidal solution of Au seeds under vigorous stirring at 150 °C, and the mixture was kept at this temperature for 2 h, resulting in the formation of core-shell Au@Pd nanoparticles in solution. After the reaction, the core-shell Au@Pd nanoparticles labeled as Au@Pd-II were purified by precipitation with methanol, followed by centrifugation and washing with methanol, then re-dispersed in 10 mL of toluene.

### Characterizations of nanoparticles

Transmission electron microscopy (TEM), high-resolution TEM (HRTEM), and scanning TEM (STEM) were performed on a JEOL JEM-2100 and JEOL JEM-2010F electron microscope operated at 200 kV with the supplied software for automated electron tomography. For the TEM measurements, a drop of the nanoparticle solution was dispensed onto a 3-mm carbon-coated copper grid, and excessive solution was removed by an absorbent paper. Then the sample was dried under vacuum at room temperature. An energy dispersive X-ray spectroscopy (EDX) analyzer attached to the TEM operated in the STEM mode was used to analyze the chemical compositions of the synthesized nanoparticles. UV-visible spectra of Ag, Au, core-shell Au@Ag, core-shell Au@Ag/Pd, core-shell Au@Pd-I and core-shell Au@Pd-II colloidal solutions were obtained on a Hitachi U-3900 spectrophotometer. Powder X-ray diffraction (XRD) patterns were recorded on a Bruker D8 diffractometer using Cu K_α_ radiation (λ = 0.154056 nm). X-ray photoelectron spectra (XPS) were collected using a Thermo Scientific K-Alpha XPS spectrometer.

### Electrochemical measurements

Electrochemical measurements were carried out in a standard three-electrode cell, which was connected to a Bio-logic VMP3 (with EC-lab software version 9.56) potentiostat. A leak-free Ag/AgCl (saturated with KCl) electrode was used as the reference. The counter electrode is a platinum mesh (1 × 1 cm^2^) attached to a platinum wire.

For the loading of the catalyst on Vulcan XC-72 carbon support, a calculated amount of carbon powder was added to the toluene solution of Au@Pd-I and Au@Pd-II colloidal solutions in toluene, respectively. After vigorously stirring the mixtures for 6 h, the Au@Pd-I/C and Au@Pd-II/C (20 wt % Pd on carbon support) were collected by centrifugation and washed thrice with ethanol, followed by drying at room temperature in vacuum.

The working electrode was a thin layer of Nafion-impregnated catalyst cast on a vitreous carbon disk. This electrode was prepared by ultrasonically dispersing 5 mg of the nanoparticles/C in 1 mL of ethanol containing 0.05 mL of Nafion solution. A calculated volume of the ink was dispensed onto the 5 mm glassy carbon disk electrode to produce a nominal catalyst loading of 51 μg cm^−2^ (Pd basis). The carbon electrode was then dried in a stream of warm air at 70 ^°^C for 1 h.

Electrochemical CO stripping voltammograms used to determine the electrochemically active surface areas (ECSAs) of the catalysts were obtained by the oxidation of pre-adsorbed CO (CO_ad_) in 0.1 M HClO_4_ at a scan rate of 50 mV s^−1^. CO was introduced into 0.1 M HClO_4_ for 20 min to allow for complete adsorption of CO onto the catalyst. During this process, the working electrode was maintained at 0.15 V. Excessive CO in the electrolyte was then purged out using N_2_ with high purity for 20 min. The amount of CO_ad_ was measured by integration of the CO_ad_ stripping peak, and corrected for electric double-layer capacitance. The specific ECSA was calculated based on the following:


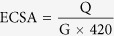


where Q is the charge of CO desorption-electrooxidation in microcoulomb (μC), which is calculated by dividing the scan rate with the integral area of CO desorption peak. G represents the total amount of Pd (μg) on the electrode, and the number (420) is the charge (μC cm^−2^) required to oxidize a monolayer of CO on the catalyst.

The performance of carbon-supported Au@Pd-I and Au@Pd-II nanoparticles in room temperature ORR was evaluated in 0.1 M HClO_4_ electrolyte solution using a glass carbon rotating disk electrode (RDE) at a rotation rate of 1600 rpm. Negative-going linear sweep voltammograms were recorded from 0.8 to 0.1 V at 10 mV s^−1^ at room temperature in the presence of bubbling ultra-pure oxygen to maintain a saturated oxygen atmosphere near the working electrode. In the ORR polarization curve, for each catalyst (Au@Pd-I/C, Au@Pd-II/C and commercial Pd/C), the measured current density was normalized in reference to ECSA of the catalysts to obtain the specific activities.

## Additional Information

**How to cite this article**: Chen, D. *et al.* Core-shell Au@Pd nanoparticles with enhanced catalytic activity for oxygen reduction reaction *via* core-shell Au@Ag/Pd constructions. *Sci. Rep.* **5**, 11949; doi: 10.1038/srep11949 (2015).

## Supplementary Material

Supplementary Information
