# Supplementary material for: Core-shell Au@Pd nanoparticles with enhanced catalytic activity for oxygen reduction reaction via core-shell Au@Ag/Pd constructions
Source: Sci Rep. 2015 Jul 6;5:11949. doi: 10.1038/srep11949 (PMC4491719; doi:10.1038/srep11949)
Supplement: Supplementary Information [file srep11949-s1.doc]

Supplementary Information

**Core-shell Au@Pd nanoparticles with enhanced catalytic activity for oxygen reduction reaction *via* core-shell Au@Ag/Pd constructions**

Dong Chen^1,2^, Chengyin Li^1,2^, Hui Liu^1^, Feng Ye^1^ & Jun Yang^1^,***

^1^State Key Laboratory of Multiphase Complex Systems, Institute of Process Engineering, Chinese Academy of Sciences, Beijing 100190, China. Fax: 86-10-8254 4814; Tel: 86-10-8254 4915; E-mail: [jyang@ipe.ac.cn](mailto:jyang@mail.ipe.ac.cn)

^2^University of Chinese Academy of Sciences, No. 19A Yuquan Road, Beijing 100049, China

Financial support from the 100 Talents Program of the Chinese Academy of Sciences and National Natural Science Foundation of China (No.: 21173226, 21376247, 21476246) is gratefully acknowledged.

**Figure S1  Au seed particles.** TEM image (a) and HRTEM image (b) of the as-prepared Au seed particles with an average diameter of 11.3 nm.

**Figure S2  Core-shell Au@Ag nanoparticles.** TEM image (a), HRTEM image (b), STEM image (c), STEM-Line scan analysis (c,d) and STEM-EDX analysis (c,e) of core-shell Au@Ag nanoparticles prepared in oleylamine using Au nanoparticles as seeds.

**Figure S3  Optical properties.** UV-Visible spectra of as-prepared colloidal solution of Au seeds, Ag nanoparticles, core-shell Au@Ag, core-shell Au@Ag-Pd, core-shell Au@Pd-I, and core-shell Au@Pd-II nanoparticles.

**Figure S4  Crystal structure of core-shell Au@Pd products and related intermediates.** X-ray diffraction (XRD) patterns of core-shell Au@Ag, Au@Ag-Pd, Au@Pd-I, and Au@Pd-II nanoparticles. The references for face centered cubic Au, Ag and Pd crystals (JCPDS Card File 040784, 040783 and 870643, respectively) are also displayed. Insert is the local amplification of the XRD patterns.

**Figure S5  Core-shell Au@Pd-II nanoparticles.** TEM image (a), HRTEM image (b) and EDX analysis (c) of core-shell Au@Pd-II nanoparticles prepared by direct growth of Pd on the Au seeds.

**Figure S6  CO stripping tests.** Cyclic voltammograms for the CO stripping on core-shell Au@Pd-I (a), core-shell Au@Pd-II (b), and commercial Pd/C-JM catalyst (c) in 0.1 M HClO_4_ at scan rate of 50 mV s^-1^. Black line: 1^st^ scan; red line: 2^nd^ scan.
